# Neutropenia and Large Granular Lymphocyte Leukemia: From Pathogenesis to Therapeutic Options

**DOI:** 10.3390/cells10102800

**Published:** 2021-10-19

**Authors:** Giulia Calabretto, Antonella Teramo, Gregorio Barilà, Cristina Vicenzetto, Vanessa Rebecca Gasparini, Gianpietro Semenzato, Renato Zambello

**Affiliations:** 1 Department of Medicine, Padua University School of Medicine, 35129 Padua, Italy; giulia.calabretto@gmail.com (G.C.); antonella.teramo@unipd.it (A.T.); gregorio.barila@gmail.com (G.B.); cristina.vicenzetto@gmail.com (C.V.); vanessarebecca.gasparini@gmail.com (V.R.G.); 2 Veneto Institute of Molecular Medicine, 35129 Padova, Italy

**Keywords:** neutropenia, large granular lymphocytes, STAT3, immunosuppressive therapy

## Abstract

Large granular lymphocyte leukemia (LGLL) is a rare lymphoproliferative disorder characterized by the clonal expansion of cytotoxic T-LGL or NK cells. Chronic isolated neutropenia represents the clinical hallmark of the disease, being present in up to 80% of cases. New advances were made in the biological characterization of neutropenia in these patients, in particular *STAT3* mutations and a discrete immunophenotype are now recognized as relevant features. Nevertheless, the etiology of LGLL-related neutropenia is not completely elucidated and several mechanisms, including humoral abnormalities, bone marrow infiltration/substitution and cell-mediated cytotoxicity might cooperate to its pathogenesis. As a consequence of the multifactorial nature of LGLL-related neutropenia, a targeted therapeutic approach for neutropenic patients has not been developed yet; moreover, specific guidelines based on prospective trials are still lacking, thus making the treatment of this disorder a complex and challenging task. Immunosuppressive therapy represents the current, although poorly effective, therapeutic strategy. The recent identification of a STAT3-mediated miR-146b down-regulation in neutropenic T-LGLL patients emphasized the pathogenetic role of STAT3 activation in neutropenia development. Accordingly, JAK/STAT3 axis inhibition and miR-146b restoration might represent tempting strategies and should be prospectively evaluated for the treatment of neutropenic LGLL patients.

## 1. Introduction

Large granular lymphocyte leukemia (LGLL) is a rare lymphoproliferative disorder characterized by the clonal expansion of cytotoxic T-large granular lymphocytes (T-LGL) or natural killer (NK) cells [1]. According to the 2016 revision of the World Health Organization’s classification of lymphoid neoplasms [2], LGLL is included among T/NK cell neoplasms, with a reported incidence of 0.2–0.72 cases per 1 million individuals per year, based on the American and Dutch registries, respectively [3,4].

T-LGL leukemia (T-LGLL) and the chronic lymphoproliferative disorder of NK cells (CLPD-NK) represent the most common subsets of the disease, accounting for ~85% and ~10% of cases, respectively [5]. Both disorders share common molecular alterations, such as the activation of several pro-survival signaling pathways and somatic mutations in the signal transducer and activator of transcription (*STAT*) genes [6,7].

Although heterogeneous, the clinical course of the disease is typically characterized by neutropenia, which turns out to be the main clinical feature, being present in up to 80% of patients along the disease course, with severe neutropenia characterizing 17–24% of cases [1,7]. Other relevant manifestations include anemia and thrombocytopenia, reported in 13–49% and 6–30% of cases, respectively [1,8,9,10,11].

Being that neutropenia is the clinical hallmark of LGLL, great efforts have been devoted to understanding its pathogenesis, in contrast with anemia or other clinical manifestations, which still remain less investigated.

Herein, we will discuss the putative pathogenetic hypotheses and mechanisms accounting for neutropenia development, particularly the recent identification of a microRNA(miR)-146b defective expression in leukemic T-LGL [12]. Moreover, we will focus on both current therapies and novel promising strategies for the treatment of neutropenic LGLL patients.

## 2. Immunological Deregulations in LGLL Patients

LGLL could be ideally placed at the intersection between a clonal lymphoproliferative disorder, autoimmunity and chronic inflammation [13]. The pathogenesis of LGLL is recognized as a multi-step process, with a chronic antigenic pressure (likely a self or viral peptide) being the triggering event for LGL activation and expansion [7]. A close topographic distribution between leukemic and dendritic cells (DC) was also described in bone marrow (BM) of patients, thus suggesting a putative role of DC in antigen presentation [14]. In turn, a strong pro-inflammatory environment plays a crucial role in LGLL pathogenesis: several cytokines and chemokines (e.g., IL-2 [15], IL-6 [16], IL-15 [17,18], IL-18, RANTES [19], PDGF [20]) are increased in sera of patients as compared to the controls, promoting LGL survival and proliferation. Of note, IL-6 was shown to be highly expressed by the non-leukemic fraction of peripheral blood mononuclear cells, thus supporting a role of other immune cell deregulations in the pathogenesis of the disease [16].

**Figure 1:**
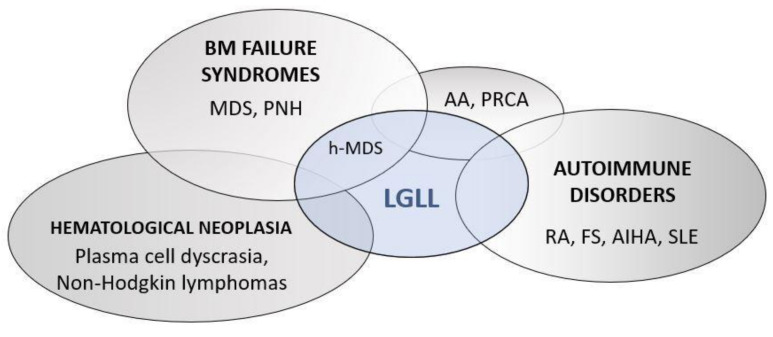
Immuno-pathogenetic background of LGLL. The Euler–Venn diagrams show the intersection between LGLL, BM failure syndromes, hematological and autoimmune diseases, emphasizing the immune and pro-inflammatory background of LGLL. AA, aplastic anemia; AIHA, Auto immune hemolytic anemia; FS, felty syndrome; h-MDS, hypocellular myelodysplastic syndrome; MDS, myelodysplastic syndrome; PNH, paroxysmal nocturnal hemoglobinuria; PRCA, pure red cell aplasia; RA, rheumatoid arthritis; SLE, systemic lupus erythematosus.

Consistently, a wide spectrum of immunological alterations (e.g., rheumatoid factor, anti-nuclear antibodies, anti-neutrophil antibodies or direct coombs) are described in LGLL patients, emphasizing the immune background of the disease [21]. A peculiar association of LGLL with autoimmune disorders is also reported, notably rheumatoid arthritis (RA), occurring in 11–36% of cases [22]. Other hematological diseases and BM failure syndromes, such as myelodysplastic syndrome (MDS), paroxysmal nocturnal hemoglobinuria (PNH), aplastic anemia (AA) and pure red cell aplasia (PRCA) frequently co-occur with LGLL (Figure 1), hinting at a widespread dysfunction of the immune system in these patients [21].

## 3. Neutrophils Lifespan and Neutropenia

Neutrophils (or polymorphonuclear leukocytes, PMN) are crucial effectors of the immune system that can be rapidly recruited to the infection sites, providing a first line of defense against pathogens. Neutrophils are produced in the BM at a rate of 10^11^ cells/day and, in physiological conditions, they are the most represented human white blood cells. This large-scale production is counterbalanced by their short life span in the bloodstream (less than 24 h) [23,24].

PMN clearance can be achieved through different mechanisms, including apoptosis and non-conventional pathways, i.e., phagocytosis-induced cell death (PICD) and autophagy. Neutrophil apoptosis can be promoted by extracellular signals (extrinsic pathways) or mitochondrial-related alterations (intrinsic pathways). In detail, extrinsic apoptotic pathways are triggered by the engagement of death receptors belonging to the tumor necrosis factor (TNF) superfamily by their respective ligands, i.e., Fas ligand (FasL), TNF-α and TNF-related apoptosis inducing ligand (TRAIL) [25,26].

In order to maintain the immune-homeostasis and to avoid the development of infectious or inflammatory diseases, the production and turnover of PMN must be finely regulated. The normal value of the absolute neutrophil count (ANC) ranges between 1.5 to 7 × 10^9^/L, with different thresholds based on age, sex and ethnicity. Neutropenia, commonly defined by an ANC < 1.5 × 10^9^/L, is classified as mild (ANC > 1 × 10^9^/L), moderate (ANC > 0.5 × 10^9^/L and < 1 × 10^9^/L), or severe (ANC < 0.5 × 10^9^/L); moreover, it can be transient or chronic, i.e., characterized by a reduction of the ANC for at least three months [27]. Chronic neutropenias can be ascribable to a congenital defect, as in the case of cyclic neutropenia, an autosomal dominant disease characterized by regular fluctuations of the neutrophil count. Differently, they can be consequent to an acquired issue and are further distinguished in primary (unknown etiology), secondary (related to an underlying disease) and drug-induced (occurring as a consequence of drug administrations). In detail, secondary acquired neutropenias can be associated with hematological diseases, such as in LGLL patients, as well as with autoimmune diseases, solid tumors or primary immunodeficiency syndromes [28,29].

## 4. Pathogenetic Hypotheses of LGLL-Related Neutropenia

**Figure 2:**
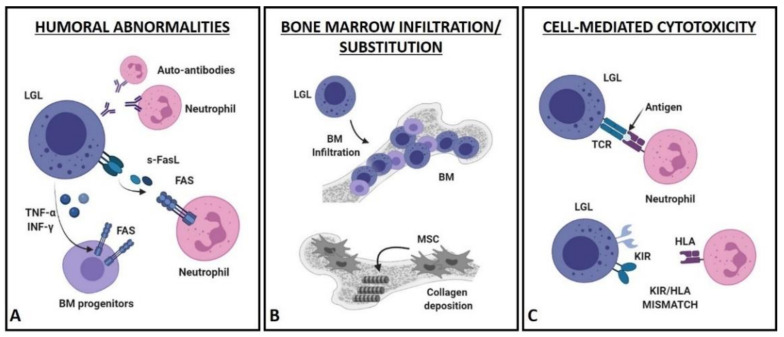
Pathogenetic mechanisms of neutropenia. (**A**) Humoral abnormalities include anti-neutrophil antibodies and the release of soluble FasL, TNF-α and ΙFN-γ by leukemic cells. Fas–FasL interaction triggers neutrophils apoptosis. TNF-α and ΙFN-γ, instead, are supposed to promote Fas expression on BM myeloid progenitors. (**B**) BM infiltration by leukemic LGL or BM substitution, due to an excessive collagen deposition by MSC, might lead to an impaired granulopoiesis. (**C**) The expression of peculiar TCR/KIR on leukemic cells surface might be responsible of a cell-mediated cytotoxicity against neutrophils. BM: Bone marrow; HLA: human leukocyte antigens; ΙFN-γ: interferon-γ; KIR: killer immunoglobulin-like receptors; LGL: large granular lymphocytes; MSC: mesenchymal stromal cells; s-FasL: soluble Fas ligand; TCR: T-cell receptor; TNF-α: tumor necrosis factor α.

The pathophysiological mechanisms underlying a chronic acquired neutropenia are various, ranging from an impaired granulopoiesis to a shortened survival of myeloid progenitors or mature neutrophils. According to the biological heterogeneity of LGLL, a unique molecular mechanism accounting for the development of neutropenia has not yet been identified, thus its pathogenesis has long been reported as a multifactorial process [29,30,31]. Three main mechanisms, alone or concurrent, were identified to explain LGLL-related neutropenia and can be classified in humoral abnormalities, BM infiltration/substitution and cell-mediated cytotoxicity (Figure 2).

### 4.1. Humoral Mechanisms (Figure 2A)

A wide spectrum of serological abnormalities is described in LGLL patients, suggesting that inflammatory or autoimmune mechanisms might play a significant role in the clinical manifestations of the disease. In particular, the finding of anti-neutrophils antibodies in sera of neutropenic patients would support an immune-mediated destruction of PMN. However, several studies failed to detect them or restricted this mechanism to a discrete subset of cases [32,33,34,35,36,37,38].

Otherwise, high levels of soluble Fas ligand (s-FasL) are found in sera of almost all neutropenic patients, as compared to patients with normal ANC and healthy controls [39,40,41]. The constitutive expression of FasL represents a peculiar feature of leukemic LGL, differently from normal cytotoxic lymphocytes, in which FasL expression occurs upon activation [42,43]. The shedding of membrane-bound FasL by ADAMs (i.e., proteins with a disintegrin and metalloprotease domain) allows the release of a soluble form of FasL (s-FasL); in turn, s-FasL is able to trigger neutrophil apoptosis via the extrinsic pathway. Consistent with a pathogenetic role of s-FasL, sera of LGLL patients were shown to induce in-vitro apoptosis of normal neutrophils; moreover, the clinical resolution of neutropenia in treated patients was associated with a reduction in s-FasL levels, which in most of cases resulted even undetectable [40,44]. PMN were also shown to be more sensitive to Fas-induced apoptosis as compared to other Fas-expressing cells, such as monocytes and eosinophils [25,45], supporting the finding of an isolated cytopenia in LGLL patients.

In physiological conditions, Fas receptor is not expressed by hematopoietic progenitors, suggesting that Fas-mediated apoptosis might be a pathogenetic mechanism restricted to mature neutrophils. However, an upregulation of Fas on CD34+ cells was demonstrated both in vitro, after stimulation with TNF-α and interferon-γ (ΙFN-γ), and in vivo in patients with aplastic anemia (AA), which are characterized by an overexpression of these cytokines [46,47]. Since leukemic T-LGL are able to spontaneously produce INF-γ and to secrete TNF-α upon stimulation, a cytokine-related increase of Fas expression on CD34+ BM precursors is likely to occur in BM of neutropenic LGLL patients, leading to a Fas-mediated apoptosis of myeloid or erythroid progenitors [48,49,50]. This hypothesis, however, does not explain the higher frequency of LGLL patients with isolated cytopenia as compared to the broad suppression of hematopoietic cells occurring in AA. In addition, the expression of Fas on granulocyte precursors was never addressed, thus the potential involvement of TNF-α and ΙFN-γ still remains to be elucidated.

### 4.2. Bone Marrow Infiltration/Substitution (Figure 2B)

Only few reports have taken into account the peculiar BM appearance of LGLL patients, mostly describing a moderate or marked hypercellularity and an interstitial lymphoid infiltration [51,52,53], with clusters of at least eight CD8+/TIA1+ cells or six granzyme B+ lymphocytes being a common histopathological finding [54]. A typical feature observed in BM of neutropenic patients is the decrease in granulocyte precursors, combined with a left-shift myeloid maturation (i.e., an increase in immature myeloid precursors in peripheral blood). An impairment of granulopoiesis is unlikely to occur, since T-LGL from patients did not show a suppressive activity towards neutrophil precursors in vitro [55]. Differently, BM invasion by clonal LGL might impair the replicative potential of hematopoietic stem cells and progenitors. To date, this issue still remains controversial, since a correlation between the percentage of BM infiltration and the degree of neutropenia (or anemia) was never demonstrated.

A role in neutropenia development could be ascribed also to reticulin fibrosis, usually increased in BM of LGLL patients, ranging from grade 2 to 3 in 50–60% of cases. Consistent with this hypothesis, patient-derived mesenchymal stromal cells (MSC) have shown an abnormal collagen matrix deposition that might interfere with the proliferation of hematopoietic progenitors or promote their premature mobilization [56]. This evidence, however, would not fully explain the high frequency of isolated neutropenia typically observed in these patients, since this mechanism might also support anemia development.

### 4.3. Cell-Mediated Cytotoxic Mechanisms (Figure 2C)

T and NK cells are characterized by a remarkable phenotypic heterogeneity, based on the variable expression of antigens and receptors, with many of them being members of the killer immunoglobulin-like receptor (KIR) family. The KIR repertoire is highly heterogeneous in the human population; in contrast, LGLL patients show a restricted pattern of KIR, mainly characterized by their activating isoforms, representing a typical feature of the leukemic clone [57,58,59]. Of note, the expression of specific KIR represents a relevant immunogenetic factor in various BM failure syndromes, including PHN, AA and PRCA [60,61]. NK cell activity, indeed, is regulated by the fine tuning of signals coming from inhibitory and activating KIR with their related ligands (i.e., the human leukocyte antigens—HLA). A bias towards the expression of activating KIR, together with a lack of inhibitory signals, might play a role in LGLL-related neutropenia or anemia [50]. To date, the main relevant finding in LGLL patients is the significant occurrence of KIR3DL2/HLA-A3/11 and KIR2DS1/HLA-C2 mismatches, which might result in the activation of a greater cytotoxic response or self-intolerance [60,62].

In addition to the possible KIR expression, T-LGL are also equipped with a T-cell receptor (TCR). A high degree of similarity between multiple immunodominant TCR clonotypes is described in LGLL patients, suggesting a non-random clonal selection [63]. Thus, the TCR specificity of leukemic clone might be involved in neutropenia development by recognizing an unknown antigen on neutrophil surface. This issue, however, is still to be addressed, since no evidence of private clonotypes or TCR-variable β (Vβ) regions, exclusively shared by neutropenic LGLL patients, has been provided so far.

## 5. Novel Insights in FasL Regulation: The STAT3/miR-146b/FasL Axis

The most credited mechanism to explain the development of neutropenia relies on the Fas-mediated apoptosis of mature neutrophils or myeloid progenitors. The Fas/FasL pathway plays a crucial role in the immune homeostasis, being involved in the activation-induced cell death (AICD) mechanism. Briefly, the engagement of Fas by FasL leads to receptor trimerization and to the recruitment of Fas-associated death domain (FADD) to Fas cytosolic portion. The complex Fas-FADD, recognized as death-inducing signaling complex (DISC), triggers the proteolytic cleavage and activation of caspase-3, ultimately leading to cell death. In LGLL, the Fas/FasL axis is known to be deregulated. Despite the abundant and constitutive expression of Fas and FasL on their surface, leukemic LGL are resistant to Fas-mediated apoptosis. This resistance is related to an impaired DISC formation and the presence of elevated levels of a soluble form of Fas (s-Fas) in sera of patients that could compete for FasL binding, acting as a decoy receptor. Loss of function mutations in FAS/FASL genes, instead, have not been identified so far [64].

Recently, a significative correlation between *STAT3* activating mutations and neutropenia was recently highlighted. *STAT3* variants represent the most distinctive genetic lesions described in leukemic LGL, being present in approximately 40% of patients [7,41,65]. Of note, STAT3 activation was shown to drive FasL expression, further supporting a STAT3 pathogenetic role in neutropenia development [41]. The clinical relevance of *STAT3* mutations was also confirmed by their association with a symptomatic and treatment-requiring disease, characterized by a reduced overall survival (OS) of patients [10].

Since FasL is not a direct STAT3 target, a high throughput miRNome analysis was performed to investigate miRNA differentially expressed in patients characterized by neutropenia, as compared to those with normal ANC and healthy controls. Selected miRNAs were analyzed for correlation with STAT3 activation and ANC. Intriguingly, miR-146b resulted to be down-regulated in T-LGL of neutropenic patients, due to a hypermethylation of its promoter. Of note, a direct role of STAT3 activation was suggested in this epigenetic alteration, by promoting the DNA methyltransferase 1 (DNMT1) expression [12].

**Figure 3:**
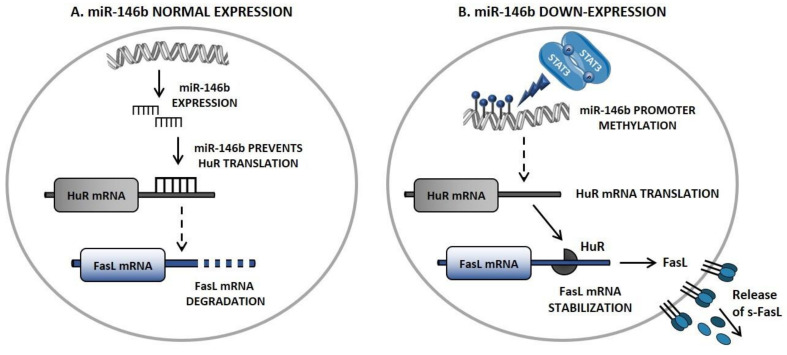
miR-146b/Fas ligand axis. (**A**) miR-146b normal expression prevents HuR translation, leading to FasL mRNA degradation. (**B**) STAT3-mediated miR-146b defective expression allows HuR mRNA translation, leading to increased FasL expression by leukemic T-LGL. FasL, Fas ligand; HuR: human antigen R; s-FasL: soluble Fas ligand; miR-146b, micro-RNA 146b; mRNA, messenger RNA; STAT3: signal transducer and activator of transcription 3.

The defective expression of miR-146b in leukemic LGL allows an increased translation of human antigen R (HuR), a RNA-binding protein and an essential FasL mRNA stabilizer. HuR-mediated FasL-mRNA stabilization leads to an increase of FasL expression, resulting in higher levels of s-FasL in sera of neutropenic patients [12] (Figure 3).

These novel data, emphasizing the role of a STAT3/miR-146b/FasL axis, elucidated the molecular mechanism accounting for the increased s-FasL expression and, for the first time, showed the involvement of an miRNA in the pathogenesis of LGLL-associated neutropenia. Other miRNAs, i.e., miR-223 [66] and miR-29b [18], are reported to be deregulated in T-LGLL patients, suggesting that transcriptional regulators (including long non-coding and circular RNAs) might play a relevant role in the pathogenetic mechanisms of the disease. 

## 6. The Prognostic Value of Immunophenotype to Identify Neutropenic LGLL Patients

Leukemic T-LGL typically shows a post-thymic effector-memory phenotype: TCR+, CD3+, CD4-, CD5^dim^, CD8+, CD27-, CD28-, CD45RA+, CD45RO-, CD57+, CD62L-, CCR7-, CD122+. Beyond the most common CD4-/CD8+ T-LGL proliferation (referred to as CD8+ T-LGLL), a CD4+/CD8^−/dim^ variant (referred to as CD4+ T-LGLL) is described in approximately 30% of cases [10]. Moreover, according to the positive or negative expression of three NK cell markers, i.e., CD16, CD56 and CD57, several immunophenotypic combinations were demonstrated in both CD8+ and CD4+ T-LGLL subsets [41].

Notably, a strong correlation between the immunophenotype of the leukemic clone and neutropenia was highlighted. Neutropenic T-LGLL patients are generally included in the CD8+ T-LGLL subset and identified by a peculiar combination of LGL markers, i.e., CD16+/CD56-. On the contrary, neutropenia is rarely observed in the other immunophenotypic subgroups (Table 1). Consistently with this observation, FasL expression was demonstrated to be higher in CD8+/CD16+/CD56- T-LGLL patients, as compared with other immunophenotypic subgroups [41].

NK cells exhibit a TCR-, CD2+, sCD3-, CD3ε+, CD4-, CD8±, CD16+, CD56+ phenotype; moreover, two main subsets can be distinguished according to CD16 and CD56 expression: CD16^dim/neg^/CD56^dim^ and CD16^bright^/CD56^dim/neg^ NK cell proliferations [67]. Neutropenic CLPD-NK patients are typically included in the CD16^bright^/CD56^dim/neg^ subgroup and characterized by the lack of expression of CD57 [68] (Table 1).

This evidence supports the added value of immunophenotypic analysis to provide relevant prognostic information. Although with some exceptions, *STAT3* mutations and/or a discrete phenotype might be useful predictors to early identify and manage this high-risk category of patients.

## 7. Differential Diagnosis of LGLL-Related Neutropenia

Neutropenic LGLL patients are typically characterized by recurrent oral ulcerations and infections, usually bacterial, involving skin, oropharynx, lung and perirectal area. Severe septic complications may also occur and represent the primary cause of related death in approximately 5–10% of cases. Acute viral and fungal infections are less common, while opportunistic infections are rarely observed [13,22,69].

Although the diagnostic work-up of neutropenia is beyond the aim of this review, there are some specific conditions that need to be considered in the differential diagnosis of LGLL-related neutropenia. LGLL diagnosis requires evidence of a chronic-expanded clonal LGL population. Peripheral blood (PB) immunophenotypic analysis, by flow cytometry, is mandatory to define LGL expansion. A threshold of at least 0.5 × 10^9^ LGL/L is now currently recognized; exceptionally, a LGL count lower than 0.5 × 10^9^/L, associated with a proper clinical context, can also be accepted [7,22]. Clonality can be assessed by TCR-γ gene rearrangement and TCR-Vβ repertoire analyses in T-LGL proliferations. As a consequence of the lack of TCR in NK cells, a restricted pattern of KIR expression is commonly used as a surrogate of clonality for NK cell expansions [7].

**Figure 4:**
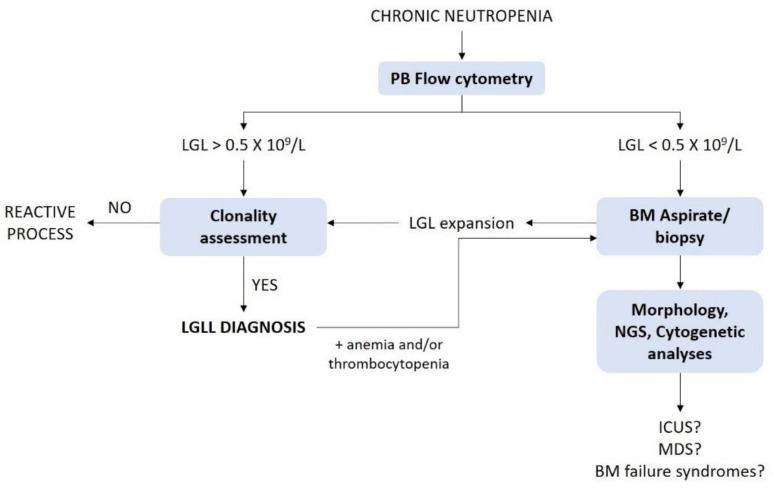
Diagnostic work-flow of LGLL-related neutropenia. BM: Bone marrow; ICUS: idiopathic cytopenia of undetermined significance; LGL: large granular lymphocytes; LGLL: large granular lymphocytes leukemia; MDS: myelodysplastic syndromes; NGS: next generation sequencing; PB: peripheral blood.

The demonstration of clonality allows to distinguish reactive from truly leukemic proliferations (Figure 4). In this regard, clonality assessment might be useful in the differential diagnosis with felty syndrome (FS), an autoimmune condition characterized by the triad of RA, neutropenia and splenomegaly. A significant number of LGLL patients, indeed, display concomitant RA. Distinguishing concomitant LGLL/RA from FS is quite challenging, since predilection of HLA-DR4 and somatic *STAT3* mutations can be detected in both conditions, supporting the hypothesis that these diseases share common pathogenetic mechanisms [30,70].

Since the diagnosis of LGLL is usually established by PB analysis, BM aspirate and/or biopsy are not routinely performed as part of the initial evaluation; BM investigation is rather recommended when the diagnosis is not straightforward, since it might help to elucidate the etiology of unexplained cytopenias [7] (Figure 4). In this regard, LGLL is frequently associated with BM failure syndrome, particularly MDS, according to a recent series showing LGL clonal expansion in approximately one-third of MDS cases [70].

Differential diagnosis is central to correctly address the therapeutic decision; in particular, alkylating agents should be avoided in the setting of concomitant myeloid neoplasms, due to the increased risk of progression to acute myeloid leukemia. In addition to neutropenia, patients with concomitant LGLL and MDS generally display anemia and/or thrombocytopenia, typical MDS-related features, such as myeloid dysplasia, cytogenetic abnormalities, increase in blasts count and the presence of clonal myeloid mutations (e.g., TET2, DNMT3A, SF3B1, ASXL1) [71,72]. Somatic mutations in *STAT3*, instead, can be detected in both concomitant LGLL/MDS and LGLL/AA patients, although with lower frequency as compared to LGLL cases [72,73].

## 8. Treatment Indications for Neutropenic LGLL Patients

LGLL patients are characterized by a heterogenous clinical course. They may remain asymptomatic for a long time, but roughly 2/3 of them develop symptoms during the natural history of the disease, with 30–70% of cases requiring therapy [50,74,75]. According to current concepts, treatment of LGLL patients is required in the presence of symptomatic or severe (ANC < 0.5 × 10^9^/L) neutropenia, symptomatic or transfusion-dependent anemia and associated autoimmune conditions [76]. In our Hematology Unit, at Padua University Hospital (Italy), asymptomatic patients, even with severe neutropenia, are not systematically treated. Recent data showed a comparable OS between treated-patients with symptomatic neutropenia and untreated-patients with severe but asymptomatic neutropenia, thus supporting our clinical approach [10].

Current conventional therapeutic strategy for LGLL patients relies on immunosuppressive therapy (IST), based on retrospective data. A targeted treatment for neutropenic patients does not exist yet and patients presenting with neutropenia share the same therapeutic options of those characterized by other clinical manifestations. Moreover, the overall response rate (ORR) and long-lasting responses are unsatisfactory, with a high number of relapsed/refractory (R/R) patients, highlighting the need for novel and effective therapeutic approaches [22].

**Figure 5:**
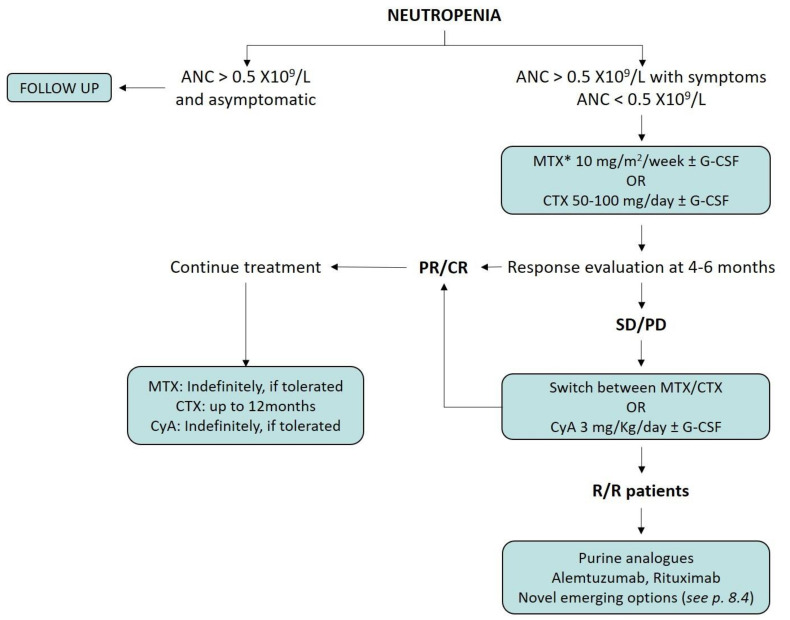
Treatment algorithm for neutropenic LGLL patients. *MTX to be preferred in *STAT3* Y640F patients; ANC: absolute neutrophil count; CR: complete response; CyA: Cyclosporine A; CTX: cyclophosphamide; G-CSF: granulocyte-colony stimulating factor; MTX: methotrexate; PD: progressive disease; PR: partial response; R/R: relapsed/refractory; SD: stable disease.

In the following paragraphs, we will discuss the available therapeutic options, as well as emerging and promising therapies for the treatment of LGLL patients, with particular attention to those presenting with neutropenia (Figure 5).

### 8.1. Immunosuppressive Therapy

First-line therapy usually relies on single-agent methotrexate (MTX, 10 mg/m^2^ per week) or cyclophosphamide (CTX, 50–100 mg per day), typically combined with steroids administration (e.g., prednisone 1 mg/kg orally daily, 30 days then gradually tapered [74]). A switch between MTX and CTX is recommended as second-line, restricting cyclosporine A (CyA, 3 mg/Kg per day) for patients failing both treatments [74,76].

Of note, a prognostic value of response to MTX was observed in patients characterized by the *STAT3* Y640F mutation [66]. Thus, MTX represents the current recommended option for neutropenic patients with this specific genetic lesion. A randomized prospective trial (#NCT01976182) comparing first-line MTX versus CTX is investigating the best therapeutic choice, also aiming at identifying other potential markers of response [22].

A minimum of 4–6 months of treatment is required before assessing response. Both MTX and CyA can be maintained indefinitely, according to their tolerance. In contrast, CTX should not be used for periods longer than 6–12 months because of its mutagenic potential [75]. Regardless of the treatment status, patients should be routinely monitored for symptoms and disease progression. Specific recommendations during the follow up include regular liver tests and chest radiographs for long-term use of MTX, while renal function and blood pressure need to be evaluated during CyA treatment [22,66,76,77,78].

### 8.2. Splenectomy and Supportive Therapy

Even though splenomegaly is described in 20–50% of patients, a relevant role of spleen-mediated destruction of neutrophils is not supported by histological findings [30,79,80]. Moreover, long-lasting responses were rarely observed in splenectomized patients, aside from a transient improvement of neutropenia. Thus, splenectomy should be limited to patients with splenomegaly-related symptoms [81].

Treatment with granulocyte-colony stimulating factor (G-CSF) may be effective in rapidly increasing the ANC. However, both splenomegaly and joint pains may get worse with prolonged G-CSF administration [13].

### 8.3. Salvage Therapies

The optimal strategy for R/R patients is difficult to assess, since prospective trials based on large cohorts of patients are limited. Purine analogs, such as fludarabine, cladribine, pentostatin and bendamustine were used in small series of R/R patients (less than 50 cases), with promising ORR (70–80%) [13].

In addition, immunotherapy with the monoclonal antibodies alemtuzumab (anti-CD52) and rituximab (anti-CD20) was considered in refractory LGLL cases. In detail, alemtuzumab showed an ORR of 74% and 47% CR rates [82]. This drug, however, should be restricted to selected cases, due to its high toxicity. Although the use of rituximab in LGLL might appear counterintuitive, it was reported to be effective in impairing leukemic cell survival and in the recovery of LGLL clinical manifestation, including neutropenia, in a patient with concomitant RA [83].

Polychemotherapy combination regimens, on the contrary, are reported to be ineffective and toxic in chronic LGL proliferations [13].

### 8.4. Emerging Therapeutic Options for LGLL Patients

According to the complex and heterogeneous pathogenesis of LGLL, several efforts were made to develop novel therapeutic approaches. In this regard, the inhibition of the JAK/STAT3 axis might represent a potential and attractive therapeutic strategy, especially for patients presenting with neutropenia. Tofacitinib citrate, a JAK3-specific inhibitor, showed its efficacy in 9 T-LGLL/RA patients, refractory to conventional therapy. A hematological response was observed in 6/9 (67%) cases, with 5 out of 7 (71%) neutropenic patients showing an increase of ANC [84]. Even with limitations due to the small cohort, tofacitinib revealed an increased efficacy in *STAT3*-mutated cases, as compared to those with wild-type *STAT3* [84].

Focusing on the pathogenetic mechanisms of neutropenia, specific alterations (e.g., miR-146b defective expression) were demonstrated to be consequent to an increased level of methylation of gene promoters [12]. Accordingly, the clinical efficacy of epigenetic modifiers should be prospectively addressed; to date, evidence on 5′-Aza-2′-deoxycytidine (a demethylating agent) [12,16] or belinostat (a histone deacetylase inhibitor) have been reported, supporting this potential therapeutic approach [85].

Another class of compounds to be considered in the therapeutic scenario of LGLL patients are proteasome inhibitors (PI), e.g., bortezomib and ixazomib, which are both approved for the treatment of multiple myeloma (MM) and various leukemias/lymphomas. These molecules are able to induce in vitro leukemic LGL apoptosis with different mechanisms, including the increase of intracellular levels of pro-apoptotic protein (e.g., Bid), commonly degraded via ubiquitination, and the inhibition of the nuclear factor k-B (NF-kB) signaling pathway [86,87]. In addition, a reduction of leukemic LGL in patients with concurrent LGLL and MM, treated with bortezomib for the plasma cell neoplasia, is documented [88]. Although this evidence is encouraging, it should be considered the potential toxicity of these therapeutic options. Thus, prospective trials are needed to investigate the efficacy and safety of these drugs in large cohorts of patients.

Among the emerging therapeutic option, there is also a multi cytokine inhibitor BNZ-1, which selectively inhibits IL-2, IL-15 and, to a lesser degree, IL-9 signaling. Previous reports have shown the effect of pro-inflammatory cytokines, including IL-15, to promote STAT3 activation [41]. A phase I/II trial (#NCT03239392) is ongoing to test this molecule in LGLL patients, with promising expectations [89].

## 9. Conclusions and Future Directions

Chronic isolated neutropenia is the clinical hallmark of LGLL patients and the most common indication for treatment. In this paper, we described a heterogeneous picture of the disease, suggesting that different subsets of patients might be characterized by discrete features and abnormalities accounting for neutropenia development. According to current evidence [7,41,68], immunophenotypic and *STAT3* mutational analyses are useful in the early identification of neutropenic LGLL patients. Moreover, this longitudinal relationship might be helpful to manage patients during the natural history of the disease.

By contrast, treatment still remains a challenging task, mainly based on empirical approaches. A targeted treatment and specific guidelines are not available, with IST being the conventional, but poorly effective, therapeutic strategy. Moreover, data on other therapeutic options are erratic and limited to small series, emphasizing the lack of effective therapies, which still represents an unmet clinical need.

We previously focused on miR-146b-FasL axis, according to the recent description of its key role in neutropenia development in T-LGLL patients [12]. Even if it could be tempting to identify FasL as a new therapeutic target for neutropenic LGLL patients, the neutralization of this pivotal signaling molecule may lead to severe side effects, that is a strong limitation. Differently, miR-146b might represent an ideal and promising candidate for the development of an innovative targeted therapy, being its defective expression a molecular signature of neutropenic patients. New advances regarding miRNA-based therapies was achieved, either based on miRNA mimics to restore lost endogenous miRNA or antagomirs to counteract the overexpression of oncogenic miRNA in cancer cells [90,91]. One of the main challenges of a miRNA-based therapy relies on the effective and selective delivery of miRNA molecules to target cells. MiR-146b restoration in leukemic T-LGL might be attained through engineered delivery systems, such as antibody-conjugated nanocarriers. Additional studies are ongoing to assess the potential role of miR-146b restoration as a novel therapeutic option for neutropenic LGLL patients.

## Figures and Tables

**Table 1:** Immunophenotypic signature of neutropenic LGLL patients.

	CD3	CD8	CD4	CD16	CD56	CD57	Neutropenia
T-LGLL	+	+	-	+	-	±	Frequent
	+	+	-	-	-	+	Rare
	+	+	-	-	+	+	Rare
	+	+	-	+	+	+	Rare
	+	+	-	-	+	-	Rare
	+	-/Dim	+	±	+	+	Rare
CLPD-NK	-	±	-	Bright	-/Dim	-	Frequent
	-	±	-	Bright	-/Dim	+	Rare
	-	±	-	Dim	Dim	±	Rare

Note: CD: Cluster of differentiation; CLPD-NK: chronic lymphoproliferative disorder of NK cells; T-LGLL: T-large granular lymphocyte leukemia.

## Data Availability

Not applicable.
